# Attenuated *Salmonella* Typhimurium expressing *Salmonella* Paratyphoid A O-antigen induces protective immune responses against two *Salmonella* strains

**DOI:** 10.1080/21505594.2018.1559673

**Published:** 2019-01-14

**Authors:** Qing Liu, Pei Li, Hongyan Luo, Roy Curtiss, Qingke Kong

**Affiliations:** a College of Animal Science and Technology, Southwest University, Chongqing, China; b Department of Infectious Diseases and Immunology, University of Florida, Gainesville, FL, USA

**Keywords:** *S*. Typhimurium, *S*. Paratyphi A, Paratyphoid fever, O-antigen, Recombinant attenuated *Salmonella* vaccine (RASV)

## Abstract

*Salmonella enterica* serovar Paratyphi A is the main causative agent of paratyphoid fever in many Asian countries. As paratyphoid is spread by the fecal-oral route, the most effective means of controlling *S*. Paratyphi A infection is through the availability of clean water supplies and working sanitation services. Because sanitation facilities improve slowly in these poor areas and antibiotic resistance is severe, the development of a safe and effective vaccine remains a priority for controlling the spread of paratyphoid disease. In this study, we investigated the strategy of heterologous O-antigenic O2 serotype (*S*. Paratyphi A characterized) conversion in *S*. Typhimurium to prevent paratyphoid infections. A series of *S*. Typhimurium mutants were constructed with replacement of *abe, wzx*_B1_ and *wbaV*_B1_ genes with respective *prt-tyv*_A1_, *wzx*_A1_ and *wbaV*_A1_, and the results showed that only three genes including *prt, wbaV_A1_* and *wzx_A1_* from *S*. Paratyphi A presence enable *S*. Typhimurium to sufficiently express O2 antigen polysaccharide. We also constructed a series of live attenuated *S*. Typhimurium vaccine candidates expressing heterologous O2 O-antigens, and a mouse model was used to evaluate the immunogenicity of live vaccines. ELISA data showed that vaccine candidates could induce a comparatively high level of *S*. Paratyphi A and/or *S*. Typhimurium LPS-specific IgG and IgA responses in murine model, and IgG2a levels were consistently higher than IgG1 levels. Moreover, the functional properties of serum antibodies were evaluated using *in vitro* C3 complement deposition and opsonophagocytic assays. Our work highlights the potential for developing *S*. Typhimurium live vaccines against *S*. Paratyphi A.

## Introduction

*Salmonella enterica* serovars Typhi (*S*. Typhi) and Paratyphi (*S*. Paratyphi A, B, and C) cause a serious systemic infection with high morbidity and mortality in humans [1]. The global burden of enteric fever is significant, with 21.6 million illnesses and 216,500 deaths reported in 2000 worldwide [2,3]. Previously, *S*. Typhi was believed to be a major cause of enteric fever. It is now becoming clear that *S*. Paratyphi A is beginning to replace *S*. Typhi as the primary causative agent of enteric fever in many Asian countries [4–7]. The highest incidence in Asia was documented in China, ranging from 0.08 to 192.5 cases per 100,000 persons per year [5]. The diagnosis of paratyphoid fever is difficult as patients generally present with non-specific febrile illness, and the treatment of *S*. Paratyphi A has been complicated in recent years due to increasing antibiotic resistance [7]. Since paratyphoid is spread by the fecal-oral route, the most effective means of controlling *S*. Paratyphi A is through the availability of clean water supplies and working sanitation services. Considering that these infrastructures tend to improve slowly, the development of a safe and effective vaccine remains a good choice for controlling the spread of paratyphoid disease [6,7].

Currently, there are no licensed vaccines for prevention of *S*. Paratyphi A infection. While research and development of *S*. Typhi vaccines is advancing, *S*. Paratyphi A vaccine development is still in an early stage. Similar to approaches in *S*. Typhi vaccine development, *S*. Paratyphi A vaccine development has been made efforts in both oral and injectable formats, i.e. whole-cell live attenuated vaccines and O-antigen polysaccharide (O2 serotype) conjugated to a range of protein carriers. The first polysaccharide vaccine against *S*. Paratyphi A in humans was conjugated to tetanus toxoid (O:2-TT), and found to be both safe and immunogenic in Phase 1 and 2 trials [8]. Following the similar strategy, dipheteria toxoid (DT) and CRM197 (non-toxic DT mutant) were used as carrier proteins to develop similar O2-specific polysaccharide conjugate vaccines [9–11]. Live oral vaccines induce the full arms of immune responses, including long-term memory and mucosal immune responses. The *phoPQ* attenuation was first applied to develop *S*. Paratyphi A live vaccine candidates and was found to be reactogenic in a dose-dependent manner [12]. Meanwhile, an oral live attenuated vaccine candidate, CVD 1902, by deleting *gua*BA and *clp*X from the wild-type *S*. Paratyphi A strain ATCC9150, induced high serum IgG responses to *S*. Paratyphi A and is now undergoing Phase I clinical evaluation [13,14]. For live vaccine candidates, there has long been a bottleneck for an oral animal model developed specifically for the human host-restricted pathogens *S*. Typhi and *S*. Paratyphi A, which has greatly retarded vaccine preclinical research. Therefore, we investigated an alternative strategy to design potential live vaccines against *S*. Paratyphi A by heterologously expressing O2-specific O-polysaccharide in *S*. Typhimurium, a host generalist.

The major distinction between the O-antigen structures of *S*. Paratyphi A (serogroup A1) and *S*. Typhimurium (serogroup B1) is their O-unit side-branch dideoxyhexoses, namely, O2 (serogroup A, α-Par(1→3)Man) versus O4 (serogroup B, α-Abe(1→3)Man) [15]. Moreover, *O*-acetylation is slightly different in *S*. Typhimurium in comparison to *S*. Paratyphi A. In *S*. Typhimurium, the abequose residue is acetylated on the 2-hydroxy group, conferring the O5 serotype, while in *S*. Paratyphi A, rhamnose is partially *O*-acetylated at the C-3 position, and no antigenic epitope is recognized in association with this modification. *O*-acetylation is consistently reported to have an effect on immunological properties [16–19]. When this chemical structural variation reflects genetic variation in O-antigen gene clusters, the difference lies in genes that are involved in the biosynthesis of the nucleotide sugar precursors and the genes responsible for later O-antigen processing [20–22]. More specifically, the *abe* gene is required for final CDP-abequose synthesis in *S*. Typhimurium, while *prt-tyv*_A1_ is required for final CDP-paratose synthesis in *S*. Paratyphi A. The other nucleotide sugar precursors, UDP-galactose, GDP-mannose and GDP-rhamnose, consisting of the common trisaccharide backbone of O-polysaccharide (O12 factor) in *S*. Typhimurium and *S*. Paratyphi A, are the same. The glycosyl transferase *wbaV*_B1_ gene is required for side branch α-Abe(1→3)Man synthesis in *S*. Typhimurium, while the *wbaV*_A1_ gene is required for side branch α-par(1→3)Man synthesis in *S*. Paratyphi A. The other glycosyl transferase genes are comparatively conserved for other sugar linkages in O-antigen synthesis among *S. enterica* serovars [21]. Although most of the O-antigens in *S. enteric* serovars are synthesized through the Wzx/Wzy-dependent pathway, there are enormous sequence variations in *wzx* and *wzy*, which can be related to the diversity of O-unit structures. The Wzx translocase is widely thought to be specific only for the first sugar of the repeating O-units. However, Wzx in fact exhibits a clear, and often strong, preference for its native substrate beyond the first sugar [23–25]. Furthermore, it is assumed that the diversity of the *wzy* sequence reflects both the variation in adjacent O-unit linkage, which is synthesized by Wzy, and the variation in other parts of the acceptor O-unit [26].

In this study, our results show that *S*. Typhimurium exhibits a strong preference for abequose side-branch O-unit rather than paratose side-branch. *S*. Typhimurium with the *abe* deletion and *prt-tyv*_A1_ insertion suffered large-scale lysis when grown in Luria-Bertani (LB) medium. This deleterious effect on *S*. Typhimurium due to paratose discrimination could be ameliorated by replacing *wzx*_B1_ and *wbaV*_B1_ with *wzx*_A1_ and *wbaV*_A1_ from *S*. Paratyphi A, respectively. To decrease the deleterious effects posed by side sugar replacement on *S*. Typhimurium and balance induced immunity against the O4 and O2 epitopes on the surface of *S*. Typhimurium, a series of live vaccine candidates were designed and constructed.

## Results

### Construction of *S.* Typhimurium mutants expressing the O2 O-antigen polysaccharide

**Figure 1.**
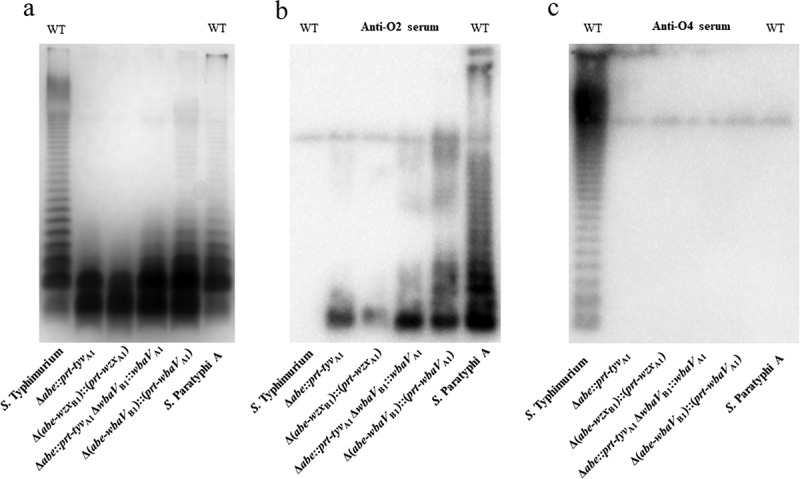
LPS profile of O2 serotype-converted mutants in *S*. Typhimurium. (a) The LPS profiles of the wild-type *S*. Paratyphi A and *S*. Typhimurium and mutants S1049 (Δ*abe*::*prt-tyv*_A1_), S1071 [Δ(*abe-wzx*_B1_)::(*prt-wzx*_A1_)], S1072 (Δ*abe*::*prt-tyv*_A1_ Δ*wbaV*_B1_:: *wbaV*_A1_) and S1074 [Δ(*abe-wbaV*_B1_)::(*prt-wbaV*_A1_)] were visualized by silver staining. (b and c) Western blot analysis confirmed the O-serotype conversion from O4 to O2. *Salmonella* O4 and O2 single-factor rabbit antisera were used as primary antibodies in the Western blot assay. Samples derived from the same experiment and gel/blots were processed in parallel.

The O-antigen gene clusters and their chemical structures of *S*. Paratyphi A and *S*. Typhimurium were shown in Supplementary Figure S1, revealing the distinction in genes, sugar components and linkages within the O-unit. *S. enterica* serogroup A1 and B1 have a common trisaccharide structure of β-Man(1→4)-α-Rha(1→3)-α-Gal with different dideoxyhexoses linked to mannose. The dideoxyhexoses contribute to their immunodominant determinants in serological specificity, namely O2 (serogroup A, α-Par-(1→3)-Man) versus O4 (serogroup B, α-Abe-(1→3) Man). To achieve synthesis of paratose side-branch O-units in *S*. Typhimurium, four mutants were constructed subsequently in case of unexpected substrate specificity. These mutant included S1049 (Δ*abe*::*prt-tyv*_A1_), S1071 [Δ(*abe-wzx*_B1_)::(*prt-wzx*_A1_)], S1072 (Δ*abe*::*prt-tyv*_A1_ Δ*wbaV*_B1_:: *wbaV*_A1_) and S1074 [Δ(*abe-wbaV*_B1_)::(*prt-wbaV*_A1_)] (Supplementary Figure S2). The O-antigen polymerase gene *wzy*_B1_ also caught our attention because of potential O-unit substrate specificity. However, the gene *wzy*_B1_ in *S*. Typhimurium shared 99% DNA sequence identities with the *wzy*_A1_ in *S*. Paratyphi A, and thus we presumed that there would be minor functional difference between *wzy*_B1_ and *wzy*_A1_. Moreover, we later showed that *wzy*_B1_ in *S*. Typhimurium had no problem in polymerizing O2 serotype O-units (Figure 1).

### Paratose side-branch O-units are inefficiently processed in *S.* Typhimurium

The silver staining and the western blotting probed by anti-O2 and O4 were performed to determine the LPS profiles of these mutants (Figure 1). The phage P22 infections were performed to further examine the biological changes due to O-antigen structure alteration, as the attachment of P22 to *Salmonella* is mediated by the binding of its tailspike protein to the O repeating units of groups B1, D1 and A1 in a different efficiency. Other phenotypes were also evaluated to investigate the effects posed by alteration of LPS in the mutants (Table 2 and supplementary Figure S3). The results showed that the strain S1049 (Δ*abe*::*prt-tyv*_A1_) produced a short LPS containing only one or two O2 antigen O-units, and its growth rate were seriously affected (Supplementary Figure S3). Compared with the wild-type *S*. Typhimurium, the number of P22 transductions obtained from its derivatives were all significantly decreased (Table 2). In accordance with that observation, the MICs of deoxycholate and polymyxin B of S1049 were eight and two-fold lower than those of the wild-type *S*. Typhimurium, respectively. Meanwhile, the swimming mobility and the virulence attribute of S1049 were all severely affected (Table 2). A similar result was also observed for the strain S1071 [Δ(*abe-wzx*_B1_)::(*prt-wzx*_A1_)]. However, unlike S1049 and S1071, the strain S1072 (Δ*abe*::*prt-tyv*_A1_ Δ*wbaV*_B1_::*wbaV*_A1_) could synthesize a better length of O-antigens, although not as well as S1074. The LPS profile from S1074 [Δ(*abe-wbaV*_B1_)::(*prt-wbaV*_A1_)] was similar to that of the wild-type *S*. Paratyphi A (Figure 1). Consistently, the MIC of deoxycholate and polymyxin B of S1071 remained as low as S1049, but S1074 was restored to the wild type level (Table 2). S1072 remained sensitive to polymyxin B when compared with the wild type. Meanwhile, we observed an improved growth rate of both S1071, S1072 and S1074 (Supplementary Figure S3). These phenotypic evaluations indicated that glycosyl transferase WbaV_B1_ in *S*. Typhimurium was ineffective in transferring CDP-paratose to the growing O2 O-unit(s) and this innate O-unit substrate specificity posed by Wzx_B1_ translocase further reinforce this ineffectiveness in synthesis of O2 polysaccharide.

**Table 1.** Bacterial strains and plasmids used in this study.

Strains or Plasmids	Description	Source
Strains		
S100	*S*. Typhimurium	[51]
S356	*S*. Paratyphi A	CDC*
S1049	Δ*abe*-1::*prt-tyv*_A1_	This study
S1071	Δ(*abe-wzx*_B1_)-1::(*prt-wzx*_A1_)	This study
S1072	Δ*abe*-1::*prt-tyv*_A1_ Δ*wbaV*_B1_1::*wbaV*_A1_	This study
S1074	Δ(*abe-wbaV*_B1_)-1::(*prt-wbaV*_A1_)	This study
S738	Δ*crp*-24 Δ*cya*-25	[35]
S1112	Δ(*abe-wbaV*_B1_)-1::(*prt-wbaV*_A1_) Δ*crp*-24 Δ*cya*-25 Δ*relA*198 Δ*pagL*7	This study
S1166	Δ*crp*-24 Δ*cya*-25 Δ*relA*198 Δ*pagL*7 Δ*asd-*16	This study
S1151	Δ*abe*-1 Δ*pagL*7::TT *araC*P_BAD_ *abe* Δ*relA*198::TT *araC*P_BAD_ *lacI* Δ*crp*-24 Δ*cya*-25 Δ*asd-*16	This study
χ7232	*E. coli endA1 hsdR17* (r_K_-, m_K_*+*) *glnV44 thi-1 recA1 gyrA relA1 ∆*(*lacZYA-argF*)*U169 λpir deoR* (φ*80dlac* ∆(*lacZ*)*M15*)	[57]
χ7213	*E. coli thi-1 thr-1 leuB6 glnV44 fhuA21 lacY1 recA1 RP4-2-Tc*::Mu λ*pir* ∆*asdA4* ∆*zhf-2*::Tn*10*	[57]
Plasmids		
pYA4278	*sacB* mobRP4 R6K *ori* Cm+	[52]
pYA3337	Asd^+^ pSC101 *ori* Ptrc	[60]
pYA3700	TT araC P_BAD_ cassette; Amp+	[59]
pSS908	Δ*abe*-1 construction	This study
pSS910	Δ*wbaV*_B1_1 construction	This study
pSS913	Δ(*abe-wzx*_B1_)-1 construction	This study
pSS929	Δ(*abe-wbaV*_B1_)-1 construction	This study
pSS241	Δ*pagL*7 construction	[63]
pSS242	Δ*relA*168 construction	[63]
pSS022	Δ*crp*-24 construction	[35]
pSS023	Δ*cya*-25 construction	[35]
pSS930	Δ*abe*-1::*prt-tyv*_A1_ construction	This study
pSS935	Δ*wbaV*_B1_1::*wbaV*_A1_ construction	This study
pSS981	Δ(*abe-wzx*_B1_)-1::(*prt-wzx*_A1_) construction	This study
pSS946	Δ(*abe-wbaV*_B1_)-1::(*prt-wbaV*_A1_) construction	This study
pSS941	Δ*pagL*7:: TT *araC*P_BAD_ *abe* construction	This study
pSS322	Δ*relA*198:: TT *araC*P_BAD_ *lacI* construction	This study
pSS978	Asd^+^, pSC101, Ptrc-(*prt-wbaV*_A1_)	This study

*CDC: Chinese center for disease control and prevention.

**Table 2.** Transduction efficiencies, MICs, swimming motility and virulence of wild type *Salmonella* and its derivatives.

		MIC			
Strain	No. of P22 transductants^a^	DOC(mg/ml)^b^	Polymyxin B(μg/ml)	Swarming motility(mm)^c^	LD_50_(CFU)
S1049 (Δ*abe::prt-tyv*_A1_)	53 ± 8 **	3.125	0.625	8.36 ± 0.82 ††	> 10^9^
S1071 [Δ(*abe-wzx*_B1_)::(*prt-wzx*_A1_)]	20 ± 4 **	3.125	0.625	6.81 ± 0.17 ††	> 10^9^
S1072 (Δ*abe::prt-tyv*_A1_ Δ*wbaV*_B1_::*wbaV*_A1_)	145 ± 34**	25	0.625	38 ± 0.24	0.92 x 10^8^
S1074 [Δ(*abe-wbaV*_B1_)::(*prt-wbaV*_A1_)]	200 ± 50 **	25	1.25	37.02 ± 0.11	1.31 x 10^7^
*S*. Typhimurium S100	587 ± 79	25	1.25	41.43 ± 1.13	1.59 x 10^5^

^a^The average numbers of chloramphenicol-resistant colonies obtained after transduction (means ± SD). Significant differences between mutant strains and the wild type S100 were indicated as “**”, *P *< 0.01.

^b^DOC, deoxycholate.

^c^The average diameter in millimeters (means ± SD). Significant differences between mutant strains and the wild-type S100 were indicated as “††”, *P* < 0.01.

### Live attenuated *S.* Typhimurium vaccines featured in O2 O-antigens

Our aim is to express heterologous O2 serotype O-antigen in *S*. Typhimurium for inducing potential protective immune responses against both *S*. Typhimurium and *S*. Paratyphi A. To achieve an excellent protection against two *Salmonella* pathogens, we designed three strategies by modifying and manipulating O-antigens on *S*. Typhimurium surface, including 1) single O2 serotype O-antigen polysaccharide synthesis, 2) O4 serotype O-antigen polysaccharide synthesis first *in vitro* and O2 serotype O-antigen polysaccharide synthesis *in vivo* latter, and 3) dual O2 and O4 serotype O-antigen synthesis. To achieve these objectives, we constructed three vaccine candidates, including S1112 [Δ(*abe-wbaV*_B1_)::(*prt-wbaV*_A1_) Δ*crp* Δ*cya* Δ*relA* Δ*pagL*], S1151 (Δ*abe* Δ*pagL*::*araC* P_BAD_ *abe* TT Δ*relA*::*araC*P_BAD_ *lacI* TT Δ*crp* Δ*cya* Δ*asd*) and S1166 (Δ*crp* Δ*cya* Δ*relA* Δ*pagL* Δ*asd*), and one plasmid, pSS978 (Supplementary Figure S2). The strain S1151 and an Asd^+^ plasmid pSS978 constituted a balanced lethal system to regulate O-antigen synthesis. When arabinose was available to *Salmonella*, LacI would be expressed from Δ*relA*::*araC *P_BAD_ *lacI* TT cassette, and prohibited P_trc_ promoter from transcribing genes *prt-tyv*_A1_, *wzx*_A1_ and *wbaV*_A1_ in the plasmid, resulting in inhibition of O2 serotype O-antigen polysaccharide synthesis. However, the normal O4 serotype O-antigen polysaccharide was synthesized because of the presence of Δ*pagL*::TT *araC* P_BAD_ *abe* on the *Salmonella* chromosome. The *abe* gene is required for abequose residue synthesis, which is responsible for the O4 serotype. When arabinose was absent during *Salmonella* growth, the S1151 carrying pSS978 would produce O2 serotype O-antigen polysaccharide, and ceased the production of O4 serotype O-antigen polysaccharide. This system developed by Dr. Curtiss was called regulated delayed antigen expression system, which was widely used to regulate protein expression [27]. Deletion mutations of *crp* and *cya* attenuated *S*. Typhimurium while retaining its O-antigen immunogenicity [28–30]. Deletion of *asd* gene, which played an essential role in peptidoglycan synthesis [31,32], resulted in obligate requirement for DAP or an Asd^+^ complementing plasmid for survival growth. The deletion of *pagL* gene limited the ability of *S*. Typhimurium to evade the immune system by lipid A 3-O-deacylation and lack of *pagL* did not alter the mutant virulence [33–35]. Δ*relA*::TT *araC* P_BAD_ *lacI* had routinely been used for arabinose-regulated LacI expression in live attenuated *S*. Typhimurium vaccine development [36].

**Figure 2.**
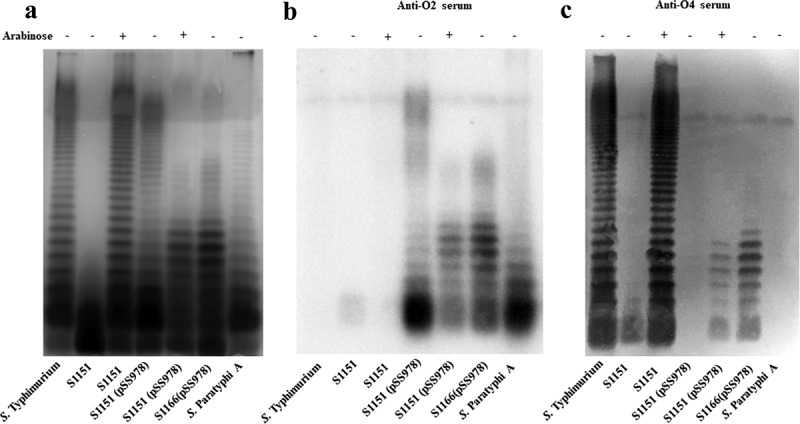
LPS profile of the live attenuated vaccine candidates S1151 (pSS978) and S1166 (pSS978). (a) The LPS profiles of the live attenuated vaccine candidates S1151 (Δ*abe* Δ*pagL*::*araC *P_BAD_ *abe* TT Δ*relA*::*araC *P_BAD_ *lacI* TT Δ*crp* Δ*cya* Δ*asd*) harboring pSS978 and S1166 (Δ*crp* Δ*cya* Δ*relA* Δ*pagL* Δ*asd*) harboring pSS978 were visualized by silver staining. Vaccine candidates were grown in medium with (+) or without (-) 0.2% arabinose, as indicated. (b and c) Western blot analysis of the LPS profile from panel A probed with anti-*Salmonella* serogroup A single-factor O2 (anti-O2) or serogroup B single-factor O4 (anti-O4) antibodies, as indicated. Samples derived from the same experiment and gel/blots were processed in parallel.

As expected, when arabinose was available *in vitro*, the strain S1151 (Δ*abe* Δ*pagL*::TT *araC *P_BAD_ *abe* Δ*relA*::TT *araC *P_BAD_ *lacI* Δ*crp* Δ*cya* Δ*asd*) produced a similar LPS pattern as the wild-type *S*. Typhimurium, but S1151 became rough in the absence of arabinose due to cease of *abe* gene expression (Figure 2(a)). S1151 harboring pSS978 generated a LPS pattern similar with *S*. Paratyphi A without arabinose in the media, but S1151 (pSS978) produced a short LPS when arabinose was available (Figure 2(a)). Compared with S1151, the strain S1166 (Δ*crp* Δ*cya* Δ*relA* Δ*pagL* Δ*asd*) did not contain a chromosomal arabinose regulation cassette for LacI expression and *abe* mutation; the P_trc_ promoter constantly transcribed the genes in pSS978. The synthesis of O-antigen polysaccharide was always observed in S1166 (pSS978) (Figure 2(a)). The western blots probed by anti-O2 and O4 sera showed that S1151 (pSS978) produced a short LPS, reacted with both anti-sera, when arabinose was available, and the similar results were also observed in S1166 (pSS978), but only O2 serotype O-antigen polysaccharide was generated in S1151 (pSS978) without arabinose (Figure 2(b,c)). S1112 [Δ(*abe-wbaV*_B1_)::(*prt-wbaV*_A1_) Δ*crp* Δ*cya* Δ*relA* Δ*pagL*] produced a same LPS containing only O2 O-antigen polysaccharides as S1074 (Δ(*abe-wbaV*_B1_)::(*prt-wbaV*_A1_) (Supplementary Figure S2).

### Evaluations of live attenuated *S.* Typhimurium vaccines

To evaluate whether heterologous expression of the O2 O-antigen in *S*. Typhimurium would influence its infection ability, we performed attachment and invasion assays and evaluated their colonization abilities in a murine model. The results showed that vaccine strains S1112, S1151 (pSS978) and S1166 (pSS978) had a significantly higher rate of attachment than their parent strain S738. Meanwhile, there was no significant difference among these vaccine strains regarding the invasion rate (Supplementary Figure S4). The colonization of murine Peyer’s patches, liver and spleen by each candidate was determined on days 4 and 8 after oral inoculation (Supplementary Figure S5). In comparison to their parent strain S738, the numbers of vaccine strain recovered from the Peyer’s patches and spleen after 8 d were significantly reduced, while the number of vaccine strains recovered from liver showed no significant differences compared to S738.

### Immunogenicity of live attenuated vaccine candidates

**Figure 3.**
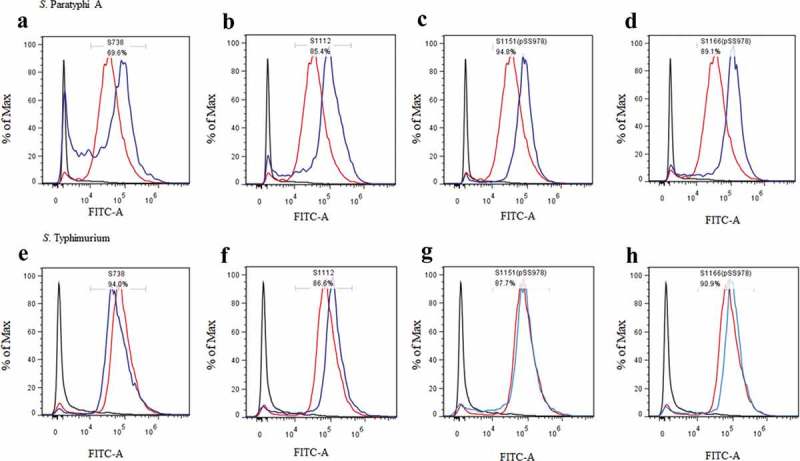
Serum IgG and mucosal IgA responses against *S*. Paratyphi A and *S*. Typhimurium LPS. The LPS of *S*. Paratyphi A and *S*. Typhimurium were used to coat ELISA plates. After a booster immunization, the serum IgG and vaginal wash IgA responses were measured by ELISA. (a) The levels of anti-*S*. Paratyphi A LPS IgG induced by S1112, S1151 (pSS978) and S1166 (pSS978) were significantly higher than those induced by S738 (***, *P *< 0.001). (b) The level of anti-*S*. Typhimurium LPS IgG induced by S738 was significantly higher than that induced by S1112 (***, *P *< 0.01), while there were no significant differences among the others. (c) Vaginal wash anti-*S*. Paratyphi A LPS IgA levels induced by S1112, S1151 (pSS978) and S1166 (pSS978) were significantly higher than those induced by S738 (***, *P *< 0.01). (D) Vaginal wash anti-*S*. Typhimurium LPS IgA induced by S738 was significantly higher than that induced by S1112 (***, *P *< 0.01). The negative control groups (BSG) did not mount a detectable immune response in any test. The concentration of antibodies was calculated using a standard curve. All concentrations of the measured samples were within the range of the standard curve. Error bars represent the standard errors of the means.

The immunogenicity of our live attenuated *S*. Typhimurium vaccine candidates was evaluated in mice. Anti-*S*. Paratyphi A and anti-*S*. Typhimurium LPS serum antibodies were measured by ELISA in triplicate and the average data were recorded. The data revealed that S1112, S1151 (pSS978) and S1166 (pSS978) induced a significantly higher anti-*S*. Paratyphi A LPS IgG immune response than S738 (Figure 3(a)). Meanwhile, there was no significant difference in anti-*S*. Typhimurium LPS IgG immune responses among S1166 (pSS978), S1151 (pSS978) and S738. However, the anti-*S*. Typhimurium LPS immune response induced by S1112 was significantly lower than S738 (Figure 3(b)). The level of the IgG isotype subclasses IgG1 and IgG2a were also examined. Consistently, all vaccines induced a significantly greater amount of IgG2a titers than IgG1 (Supplementary Figure S6). Furthermore, the IgA responses in vaginal washes were showed that all vaccines induced significantly higher anti-*S*. Paratyphi A mucosal IgA responses than their parent strains S738, and S1112 was the highest among them (Figure 3(c)). Only S1112 induced a significantly lower level of anti-S. Typhimurium mucosal IgA responses when compared with S738 (Figure 3(d)). The negative control groups (BSG) in each test did not mount a detectable immune response.

### C3 complement deposition and opsonic phagocytosis

**Figure 4.**
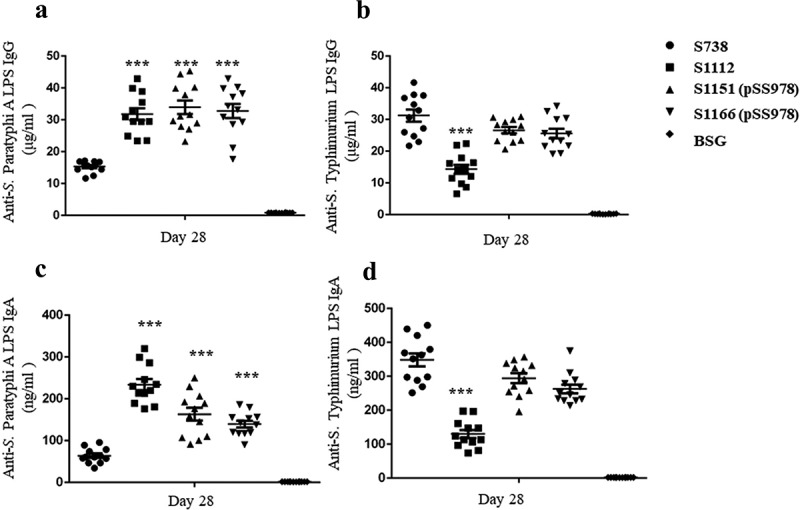
Flow cytometry histograms of C3 complement deposition. The percentage of FITC-positive bacteria was determined by flow cytometry. The wild-type *S*. Paratyphi A was incubated with the sera from mice that had been vaccinated with S738 (a), S1112 (b), S1151 (pSS978) (c) and S1166 (pSS978) (d). Accordingly, approximately 69.6%, 85.4%, 94.8% and 89.1% *S*. Paratyphi A were detected positive, respectively. The wild-type *S*. Typhimurium was incubated with sera from mice that had been vaccinated with S738 (e), S1112 (f), S1151 (pSS978) (g) and S1166 (pSS978) (h). Accordingly, approximately 94.0%, 86.6%, 87.7%, 90.9% of *S*. Typhimurium were detected positive, respectively. The blue line indicated the test groups mentioned above. The negative control (black line) was the wild-type *S*. Paratyphi A or *S*. Typhimurium incubated with naïve mice serum, and the positive control (red line) was the wild-type *S*. Paratyphi A or *S*. Typhimurium incubated with O2 or O4 single-factor rabbit antisera.

**Figure 5.**
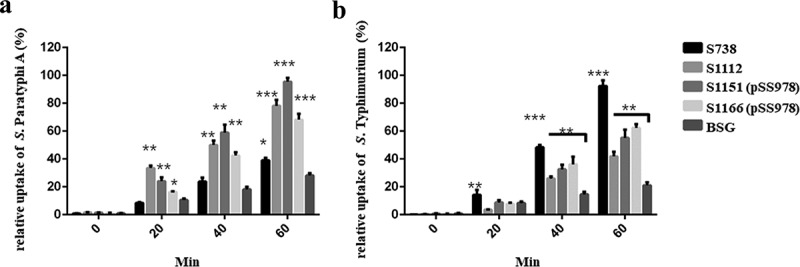
The differential uptake of *S*. Paratyphi A and *S*. Typhimurium by RAW264.7 cells after serum opsonization. Opsonophagocytic uptake of the wild-type *S*. Paratyphi A and *S*. Typhimurium by RAW264.7 macrophages was evaluated after treatment with the sera from mice that had been immunized with live attenuated vaccine candidates. (A) In comparison to the control (*S*. Paratyphi A opsonized with naïve serum), the uptake of *S*. Paratyphi A opsonized with sera from mice primed with S1112, S1151 (pSS978) and 1066 (pSS978) was significantly higher (**, *P *< 0.01). (B) The uptake of *S*. Typhimurium opsonized with sera from mice primed with S738, S1112, S1151 (pSS978) and S1166 (pSS978) was significantly higher than those opsonized with naïve mice serum (**, *P *< 0.01; ***, *P *< 0.001). The number of enumerated cells was normalized to 100% as the maximal value. The error bars represented the standard errors of the means.

C3 complement deposition is a key process leading to complement activation and later serum bactericidal activity. In addition, antibody-dependent opsonic phagocytosis is an important defence mechanism against *Salmonella* infections. To investigate the functional properties of serum antibodies, we determined the ability of sera from vaccinated and control mice to direct complement deposition on the surface of the wild-type *S*. Paratyphi A and *S*. Typhimurium and to facilitate uptake of *S*. Paratyphi A and *S*. Typhimurium by RAW264.7 macrophages. The sera used in this assay were pooled sera from mice vaccinated with S738, S1112, S1151 (pSS978), S1166 (pSS978) and the BSG control after booster. The percentage of bacteria coated with C3 was determined by flow cytometry (Figure 4). In comparison to the negative control, a high percentage of *S*. Paratyphi A showed C3 complement deposition when incubated with sera from mice immunized with S1112, S1151 (pSS978) and S1166 (pSS978). Similarly, a high percentage of *S*. Typhimurium showed C3 complement deposition on their surface when incubated with sera from mice immunized with S738, S1112, S1151 (pSS978) and S1166 (pSS978). These results indicated that antibodies in the mice sera induced by live vaccine candidates were able to trigger the classical pathway of complement activation. Furthermore, the data showed that the uptake of *S*. Paratyphi A opsonized with sera from mice vaccinated with S1112, S1151 (pSS978) and S1166 (pSS978) was significantly increased compared with mice vaccinated with the BSG control. Similarly, the uptake of *S*. Typhimurium opsonized with sera from mice vaccinated with S738, S112, S1151 (pSS978) and S1166 (pSS978) was significantly increased compared with mice vaccinated with the BSG control (Figure 5).

### Protective efficacy of live attenuated vaccines

As no animal model is available for *S*. Paratyphi A infection, we only evaluated the protective efficacy of our live attenuated vaccines against a mouse-virulent strain *S*. Typhimurium S100. The protection experiment was conducted twice. All vaccinated mice survived the 100 times LD_50_ challenge with *S*. Typhimurium with no any signs of distress and the mice in control group showed severe signs of disease, even death, indicating that our vaccine strains could provide a high level of homologous protection, even though they were converted to express the O2 epitopes (data not shown).

## Discussion

Although both oral and injectable vaccines for typhoid fever have been licensed for use for decades, there is currently no licensed vaccine against *S*. Paratyphi A for humans. As O-antigen portion of LPS is an essential virulence factor and a protective antigen, it has been speculated that antibody against the LPS of *S*. Paratyphi A would also provide protection against infection [37]. While injectable Vi polysaccharide conjugate vaccines have been intensely studied, paratyphoid O-polysaccharide conjugate vaccine development has been largely retarded. Moreover, there is a bottleneck for oral paratyphoid vaccine development due to the lack of appropriate animal models for preclinical tests. Therefore, additional efforts are needed to develop potential vaccine candidates. In this study, we constructed a series of live attenuated *S*. Typhimurium vaccine candidates expressing heterologous O2 O-antigens and proved that they could induce protective immune response to *S*. Paratyphi A.

We and others have demonstrated that *S*. Typhimurium was able to convert O4 serotype into O9, when the *abe* gene was replaced with *prt-tyv*_D1_ from *S*. Enteritidis (D1 serogroup), indicating that *S*. Typhimurium could tolerate well with tyvelose side-branch O-units [24,38,39]. However, the replacement of *abe* in *S*. Typhimurium with *prt-tyv*_A1_ from *S*. Paratyphi A did not result in a smooth LPS reacted with anti-O2 antisera. Only an O2 semi-rough LPS carrying a paratose side-branch O-unit was observed in strain S1049 (Δ*abe*::*prt-tyv*_A1_) (Figure 1) and the growth rate of this strain was severely inhibited (Supplementary Figure S3). This deficiency did not ameliorate when extra *wzx_A1_* from *S*. Paratyphi A was introduced into the strain S1071 [Δ(*abe-wzx*_B1_)::(*prt-wzx*_A1_)] (Supplementary Figure S2). In fact, the introduction of *wbaV*_A1_ from *S*. Paratyphi A into S1049 (Δ*abe*::*prt-tyv*_A1_), which is responsible for synthesis of paratose side-branch, partially restored LPS harbouring O2 epitope, and S1074 [Δ(*abe-wbaV*_B1_)::(*prt-wbaV*_A1_)], which was generated by introduction of both of *wzx_A1_* and *wbaV_A1_* from *S*. Paratyphi A into the strain S1049 (Δ*abe*::*prt-tyv*_A1_), could express O2 serotype O-antigens with no obvious defective effects (Supplementary Figure S2). All these results demonstrate that 1) the side-branch residue is required for effective O-unit translocation by Wzx_B1_ [24]; 2) WbaV_B1_ from *S*. Typhimurium has ability to transfer CDP-paratose (O2 epitope) to common chain, but its function is limited; 3) the paratose side-branch O-unit is not optimal to Wzx_B1_ from *S*. Typhimurium, but it is still capable of translocating this O-unit antigen polysaccharides across the bacterial membrane; 4) three genes including *prt, wbaV_A1_* and *wzx_A1_* from *S*. Paratyphi A will be sufficient to express O2 antigen polysaccharide in *S*. Typhimurium.

LPS is an important virulent determinant and truncation of LPS will attenuate *Salmonella* virulence [40]. In accordance, we observed that the strains S1049 (Δ*abe*::*prt-tyv*_A1_) and S1071 [Δ(*abe-wzx*_B1_)::(*prt-wzx*_A1_) exhibited significant low virulence with the LD_50_ greater than 10^9^ (Table 2), because these two strains produced a truncated LPS, resulting in severe growth defect (Figure 1 and Supplementary Figure S3). The strains S1072 (Δ*abe*::*prt-tyv*_A1_ Δ*wbaV*_B1_::*wbaV*_A1_) and S1074 [Δ(*abe-wbaV*_B1_)::(*prt-wbaV*_A1_)] producing minor smooth LPS also showed the decreased virulence, which was consistent with the notion that the expression of heterologous O-antigen polysaccharide in *Salmonella* exerted impacts on bacterial colonization and virulence [38,39,41].

The vaccine strain S1151 (Δ*abe* Δ*pagL*::TT*araC* P_BAD_ *abe* Δ*relA*::TT *araC* P_BAD_ *lacI* Δ*crp* Δ*cya* Δ*asd*) was tightly regulated to express O4 O-antigen polysaccharide by arabinose in the media (Figure 2), but it seemed that pSS978 was not tightly regulated by LacI expressed by Δ*relA*:: TT *araC* P_BAD_ *lacI* on the *Salmonella* chromosome, leading to production of a mixed O-antigen polysaccharide reacted with both anti-O2 and anti-O4 single-factor *Salmonella* antiserum. Moreover, this same mixed O-antigen polysaccharide was also observed in S1166 (Δ*relA* Δ*pagL* Δ*crp* Δ*cya* Δ*asd*) (pSS978), which constitutively expressed both O2 and O4 O-antigens (Figure 2). This result further demonstrated that P_trc_ promoter in the plasmid pSS978 harboured in S1151 was not completely shut off by LacI induced by arabinose in the media. Therefore, the vaccine strain S1151 (pSS978) expressed a mixed O-antigen polysaccharide with abequose side-branch (O4) and paratose side-branch (O2) *in vitro* when grown in the media with arabinose, and then this hybrid O-antigen would be gradually converted to the O2 serotype after the infection and invasion of hosts when arabinose was gradually consumed.

The vaccine strains S1112, S1151 (pSS978) and S1166 (pSS978) could mount significantly higher anti-*S*. Paratyphi A LPS IgG levels after a boosted vaccination than their parent S738 (Figure 3(a)). Similarly, vaccine strains S738, S1151 (pSS978) and S1166 (pSS978) could mount a high level of anti-*S*. Typhimurium LPS IgG levels with no significant differences (Figure 3(b)). Positive detection of an anti-*S*. Paratyphi A LPS immune response in the sera of mice vaccinated with S738 might have been due to the common trisaccharide backbone of the O-polysaccharide (O12 epitope) in *S*. Typhimurium and *S*. Paratyphi A (Figure 3(a)). Moreover, the consistently observed higher levels of IgG2a than IgG1 indicated that cellular immunity directed by Th1 cells was dominant for all these immune responses [42]. We further evaluated the presence of IgA in vaginal washes after a boosted vaccination. Vaccine strains S1112, S1151 (pSS978) and S1166 (pSS978) showed significantly higher levels of anti-*S*. Paratyphi A LPS IgA than S738 (Figure 3(c)), and the responses induced by S1112 was the highest among them. Similarly, vaccine strains S738, S1151 (pSS978) and S1166 (pSS978) showed high levels of anti-*S*. Typhimurium LPS IgA (Figure 3(d)). We also investigated whether the serum antibodies could enhance C3 complement deposition (Figure 4) and opsonic phagocytosis (Figure 5). The data showed that antibodies in mice sera induced by the live vaccine candidates were able to trigger the classical pathway of complement activation and promote the uptake of the wild-type *S*. Paratyphi A and *S*. Typhimurium by RAW264.7 macrophages.

As *S*. Typhimurium typically invades only local intestinal epithelial tissue and does not penetrate to the deeper tissues, attenuated *S*. Typhimurium strains are often not considered for human use. However, there have been two clinical trials in which they were evaluated. Human subjects orally immunized with attenuated *S*. Typhimurium strains LH1160 [43] and WT05 [44] developed strong mucosal or serological responses, indicating that the protection would theoretically depend largely on local immune induction sites [44–47]. Therefore, we propose that a properly attenuated *S*. Typhimurium strain could also be used as a live paratyphoid vaccine for human use.

In summary, *S*. Typhimurium was ineffective in processing paratose side-branch O-units, while this innate O-unit substrate discrimination could be overcome by replacing the *abe, wzx*_B1_ and *wbaV*_B1_ genes in *S*. Typhimurium with *prt-tyv*_A1_, *wzx*_A1_ and *wbaV*_A1_ from *S*. Paratyphi A, respectively. We found that CDP-paratose and CDP-abequose could simultaneously participate in O-units construction, which might theoretically result in a heterologous polysaccharide composed of an unclear proportion of abequose or paratose side-branch O-antigen. Moreover, a series of vaccine candidates was constructed to optimize the level of O4 and O2 O-antigenic epitopes in *S*. Typhimurium. The vaccine candidates could elicit large amounts of anti-*S*. Paratyphi A and/or anti-*S*. Typhimurium LPS antibodies, which possessed functional properties for enhancing C3 complement deposition on the surface of *S*. Paratyphi A and/or *S*. Typhimurium and induced antibody-dependent opsonophagocytosis. Our future research will try to resolve the chemical structure of the hybridized O-antigen structure and we propose a formulation of trivalent protective O-polysaccharide or oligosaccharide antigens, which constitutes an expected proportion of abequose, tyvelose and paratose side-branch O-units. This anticipated structure could be achieved by advanced technologies such as genetic engineering, synthetic biology, or chemistry synthesis.

## Materials and methods

### Bacteria, plasmids, media and growth conditions

The bacteria and plasmids used in this study are listed in Table 1. All constructed *Salmonella* mutants were derived from the virulent wild-type S. Typhimurium S100 [48]. *E. coli* and *Salmonella* were grown at 37°C in LB broth or on LB agar. Unmarked directed mutations in *Salmonella* were generated using the temperature-sensitive *sacB* gene-based counter-selectable suicide vectors [49]. When required, gene expression was regulated *via* the arabinose P_BAD_ promoter [50]. Activation of the P_BAD_ promoter was induced by 0.2% arabinose in the media. Chloramphenicol (25 µg/ml) was added for specific selection, and diaminopimelic acid (DAP) (50 µg/ml) was added for growth of the *asd* mutant strains [51]. Allelic gene exchange was performed on LB agar containing 10% sucrose with no sodium chloride and growth at 30°C. Electrocompetent *E. coli* or *Salmonella* cells were prepared as described previously [52]. *In vitro* growth rates of *Salmonella* were determined by optical density measurements.

### Molecular procedures and genetic manipulations

DNA manipulations were carried out using standard methods [53]. No restriction endonuclease site was introduced when amplifying DNA fragments from the chromosome or plasmid. The DNA concentration and purity were measured using a Nanodrop ND-2000 spectrophotometer. DNA fragments were assembled using Gibson Assembly Master Mix according to the manufacturers’ instructions (New England BioLabs). Unmarked deletion and deletion-insertion mutations in *S*. Typhimurium were constructed using sucrose counter-selectable suicide vectors. Conjugational transfer of suicide vectors to *S*. Typhimurium was performed using the suicide vector donor strain χ7213 [54]. All primers used in this study are listed in Supplementary Table 1. For deletion mutation, the up-stream and down-stream of the *abe* gene were amplified using primer pairs Dabe-1F/Dabe-1R and Dabe-2F/Dabe-2R, respectively. These two DNA fragments were then fused by PCR using the primer pairs Dabe-1F/Dabe-2R. The fused PCR products were ligated into pYA4278 through convenient T/A ligation to generate the suicide vector pSS908. The donor strain χ7213 containing pSS908 was co-cultured with *S*. Typhimurium. Successful homologous recombination with pSS908 integrated into the *S*. Typhimurium chromosome was selected on chloramphenicol agar without DAP supplementation. The second homologous recombination event, which resulted in excision of the suicide vector from the *S*. Typhimurium chromosome with either successful or abortive allelic exchange, was selected on 10% sucrose LB plates with no sodium chloride and grown at 30°C. Successful *abe* gene deletion was confirmed by PCR screening and subsequent sequencing. The same strategy was applied to generate the pSS910, pSS913 and pSS929 suicide vectors, which were used to delete genes *wbaV*_B1_, *abe-wzx*_B1_ and *abe-wbaV*_B1_, respectively. For the insertion mutations, genes *prt-tyv*_A1_ were amplified from purified *S*. Paratyphi A chromosomal DNA using primer pairs (G)In_(prt-tyvA1)-F/(G)In_(prt-tyvA1)-R. In addition, the entire backbone of the suicide vector pSS908 was amplified using primer pairs (G)Vec-Dabe-(prt-tyvA1)-F/(G)Vec-Dabe-(prt-tyvA1)-R. These two purified linear DNA fragments were assembled using the Gibson Assembly method [55], resulting in a new suicide plasmid, pSS930, for deletion of the *abe* gene and replacement by *prt-tyv*_A1_. The donor strain χ7213 containing pSS930 was used for conjugating with the *abe* deleted *S*. Typhimurium mutant, and the subsequent procedures for successful selection of the *prt-tyv*_A1_ insertion mutation were the same as described for the deletion mutation. The same strategy was applied to generate the pSS935, pSS981 and pSS946 suicide vectors, which were used to replace the original *wbaV*_B1_, *abe-wzx*_B1_ and *abe-wbaV*_B1_ genes in *S*. Typhimurium with *wbaV*_A1_, *prt-wzx*_A1_ and prt-*wbaV*_A1_, respectively. For arabinose-regulated gene expression, the entire backbone of pYA3700 carrying the TT *araC* P_BAD_ module [56] was amplified using primer pairs (G)3700-F/(G)3700-R, and the *abe* gene was amplified from purified *S*. Typhimurium chromosomal DNA using primer pairs (G)3700-abe-F/(G)3700-abe-R. These two linear DNA fragments were fused to generate a functional TT *araC* P_BAD_ *abe* TT cassette. This functional cassette was then amplified using primer pairs (G)araCP_BAD_-abe-F/(G)araCP_BAD_ -abe-R, and the entire backbone of the suicide vector pSS241 was amplified using primer pairs (G)Vec-DpagL-(araCP_BAD_-abe)-F/(G)Vec-DpagL-(araCP_BAD_-abe)-R. Again, these two linear DNA fragments were assembled to generate pSS941, resulting in the *pagL* gene deletion and insertion of the arabinose-regulated *abe* expression cassette TT *araC* P_BAD_ *abe* TT. Selection of the Δ*pagL*::TT *araC* P_BAD_ *abe* TT mutation in *S*. Typhimurium was performed as the same procedure described in insertion mutation above. The same strategy was applied to generate pSS322 and construct the Δ*relA*::TT araCP_BAD_ *lacI* TT mutation in *S*. Typhimurium.

### *In vitro* and *in vivo* regulation of *prt-tyv*_A1_, *wzx*_A1_ and *wbaV*_A1_ genes expression

To construct a regulated expression of gene cassette *prt-wbaV*_A1_, the entire backbone of pYA3337, which carried the P_trc_ promoter embedded with a *lac* repressor, was amplified using primer pairs (G)3337-F/(G)3337-R. The *prt-tyv*_A1_, *wzx*_A1_ and *wbaV*_A1_ genes were amplified from purified *S*. Paratyphi A chromosomal DNA using primer pairs (G)3337-prt-wbaVA1-F/(G)3337-prt-wbaVA1-R. These two linear DNA fragments were assembled in order to generate a new recombinant Asd^+^ plasmid, pSS978. Only the *S*. Typhimurium *asd* mutants harboring the Asd^+^ recombinant plasmids were able to grow in a sustainable manner on LB agar plates without DAP [51,57]. Plasmid pSS978 contained *prt-tyv*_A1_, *wzx*_A1_ and *wbaV*_A1_ genes under a P_trc_ promoter, which carried a lac repressor and would be repressed by LacI [27]. The expression of LacI was regulated by arabinose availability *via S*. Typhimurium mutants carrying the Δ*relA*::TT araCP_BAD_ *lacI* TT mutation. Therefore, the transcription of *prt-tyv*_A1_, *wzx*_A1_ and *wbaV*_A1_ from the P_trc_ promoter was inhibited by the addition of arabinose to the growth medium. This inhibition was relieved when the *S*. Typhimurium mutant colonized the gut-associated lymphoid tissue, where little or no arabinose was available [58]

### LPS silver staining and western blot

LPS samples were prepared, separated and visualized by the method of Hitchcock and Brown [59]. LPS samples were transferred to PVDF membranes using a Trans-Blot SD semidry transfer (Bio-Rad, Hercules, CA, USA). LPS samples were first incubated by O-antigen single-factor rabbit antisera (BD Biosciences) or vaccinated mice pooled sera (1:100 dilution) and then followed by a secondary anti-rabbit or anti-mouse alkaline phosphatase-conjugated antibody (Sigma-Aldrich, St. Louis, MO, USA) at a 1:1000 dilution. Patterns were detected by chemiluminescence using ECL western blotting Substrates (Bio-Rad, Hercules, CA, USA).

### P22 transduction studies

P22HT *int* was propagated on *S*. Typhimurium S100 carrying the integrated suicide plasmid pSS241 [60,61], which confers chloramphenicol resistance. Strains to be tested were grown overnight in LB at 37°C. Cultures were diluted 1:100 into fresh, prewarmed LB broth and grown at 37°C to an OD_600_ of 0.6. Then, 10 µl of phage (1 × 10^8^ PFU) was added to 1ml of cells (5 × 10^6^ CFU) and the mixture was incubated at room temperature for 30 min, centrifuged and resuspended in 1 ml of PBS. The 100 µl aliquot was spread onto LB agar plates containing 25µg/ml chloramphenicol and incubated overnight at 37°C. Colonies were counted the following day. This experiments were repeated three times.

### Motility assay

Motility assay was performed on LB plates solidified with 0.3% agar. The plates were allowed to dry at room temperature for about 2 h before the assays. 6 µl of freshly grown bacteria (5 × 10^6^ CFU) were spotted onto the middle of the plates, and incubated at 37°C for 6 h. The centimeter diameter of the colonies was measured. This experiments were repeated three times.

### Minimum inhibitory concentration (MIC) test

The MICs of different antimicrobial substances were determined using 96-well microtitre plates. Two-fold serial dilutions of the sodium deoxycholate (DOC) (0.39–59 mg/ml) and polymyxin B (0.078–10 µg/ml) were made down the plates. Bacteria were grown until they reached an OD_600_ of 0.8 and diluted to 1.0 × 10^6^ CFU in LB broth. Then, 100 µl of the diluted cell suspension was added to each well. The microtitre plates were incubated overnight at 37°C. The optical density of each culture was determined using an iMark^TM^ Microplate Reader (Bio-Rad, Hercules, CA, USA). The threshold of inhibition was 0.1 at OD_600_.

### Attachment and invasion assay

The Human epithelial type 2 (Hep-2) cell line (ATCC strain CCL-6) was used to perform bacterial attachment and invasion assays as described previously [40]. The percentage of attachment rate was calculated as follows: percent attachment = 100 × (number of cell-associated bacterial/initial number of bacterial added). The percentage of invasion rate was calculated as follows: percent invasion = 100 × (number of bacteria resistant to gentamicin/initial number of bacteria added). Both assays were repeated three times in triplicate.

### Virulence determination and colonization in mice

All animal research was conducted in compliance with the Animal Welfare Act and regulations stated in the Guide for the Care and Use of Laboratory Animals. The protocol was approved by Institutional Animal Care and Use Committee of the Southwest University.

Six-week-old, female BALB/c mice were purchased from Dashuo Biotechnology Co., Ltd. (Chengdu, China). For determination of the 50% lethality dose (LD_50_), bacteria were grown statically overnight at 37℃. The overnight cultures were diluted 1:100 into new fresh LB media and harvested by centrifugation at 3,452 × g at room temperature when their OD_600_ reached 0.8 to 0.9. Washed once, and normalized to the required inoculum density in buffered saline with gelatin (BSG) by adjusting the suspension to the appropriate OD_600_ value. Groups of six mice each were infected orally with 20 μl BSG containing various doses of S. Typhimurium S100 or its derivatives, ranging from 1 × 10^5^ CFU to 1 × 10^9^ CFU. Animals were observed for 4 weeks after infection, and deaths were recorded daily. The animal experiment for determining LD_50_ of each strain was performed twice and the data were combined for analysis. The LD_50_ was calculated using the method of Reed and Muench [62]. To evaluate colonization, Groups of three mice were orally inoculated with 20 μl BSG containing 1 × 10^9^ CFU vaccines. On days 4 and 8 post-inoculation, Peyer’s patches, spleen and liver samples were collected. Samples were homogenized and dilutions were plated onto MacConkey and LB agar to determine viable counts.

### Immunization and measurement of immune response

The animal experiment for determining protection rate was performed twice. For the first experiment, groups of twelve mice each were inoculated orally with 20 μl of BSG containing approximately 1 × 10^9^ CFU vaccine strains on day 0 and boosted on day 14 with the same dose. Vaginal wash samples and Blood samples were collected on day 28. Blood was obtained from mice by eye venous plexus bleeding and vaginal secretion specimen was obtained by repeated wash with 50 μl of BSG. Mice were challenged orally on day 56 with 1 × 10^7^ CFU of *S*. Typhimurium (~100 times LD_50_). For the second experiment, groups of six mice each were inoculated orally with 20 μl of BSG containing approximately 1 × 10^9^ CFU vaccine strains on day 0 and boosted on day 14 with the same dose. Mice were challenged orally on day 30 with 1 × 10^7^ CFU of *S*. Typhimurium (~100 times LD_50_).

*S*. Typhimurium LPS were purchased from Sigma (St. Louis, MO, USA). *S*. Paratyphi A LPS were purified as described previously [63]. A quantitative enzyme-linked immunosorbent assay (ELISA) was used to determine antibody titers in the serum with the following modifications. Microtiter plates were coated with *Salmonella* LPS. The capture antibody, unlabelled goat anti-mouse IgG (H + L) (BD Pharmingen, San Diego, CA) at 1 μg/ml in PBS, was added to extra uncoated wells to generate the standard curve. The plates were incubated overnight at 4°C, followed by blocking with PBS containing 10% FBS for 1 hour at room temperature. For the LPS coated wells, a 100 μl of diluted serum or vaginal washes sample was added to individual wells in triplicate. For the capture antibody-coated wells, the purified mouse IgG standard (for the standard curve quantification, BD Pharmingen, San Diego, CA, USA) was added and followed by a two-fold serially dilution starting at 0.5 μg/ml. The plates were incubated for 1 h at 37°C and then treated with biotinylated goat anti-mouse IgG (Southern Biotechnology Associates, Birmingham, AL, USA). The wells were developed with a streptavidin-alkaline phosphatase conjugate (Southern Biotechnology Associates, Birmingham, AL, USA), followed by a *p*-nitrophenylphosphate substrate (Sigma-Aldrich, St. Louis, MO, USA). Absorbance was recorded at 405 nm using an iMark^TM^ Microplate Reader (Bio-Rad, Hercules, CA, USA). The ELISA standard curve was drawn using software Curve Expert (Hyams DG, Starkville, MS, USA). Serum and vaginal secretion antibody levels were quantified based on absorbance values and the standard curve.

### Complement deposition assay

Sera used for complement deposition assays were pooled sera taken from mice after the second immunization and were heated at 56°C for 30 min to inactivate endogenous complement. Bacteria were grown to an OD_600_ of 0.8 and harvested by centrifugation at 6000 rpm for 2 min. Bacterial pellets were washed, centrifuged, and resuspended to approximately 5 × 10^8^ CFU/ml in PBS. Then, 20 µl of bacterial sample was incubated with 80 µl of complement-inactivated sera at 37°C for 30 min. Bacteria were then washed once with PBS, resuspended and incubated with 100 µl of fresh naïve BALB/c mouse sera at 37°C for 30 min. After another wash with PBS, the samples were incubated with 100 µl of FITC-conjugated goat anti-mouse complement C3c (Abcam) at a dilution of 1:100 on ice for 30 min in the dark. After the incubation, the bacteria were washed with PBS, resuspended in 1% formaldehyde, and latter analyzed with a flow cytometer (BD FACSVerse™). The negative control was the wild-type *S*. Typhimurium or wild-*S*. Paratyphi A incubated with non-vaccinated complement-inactivated mice sera, and the positive control was wild-type *S*. Typhimurium or wild-*S*. Paratyphi A incubated with complement-inactivated rabbit anti-O4 or anti-O2 *Salmonella* sera (BD Biosciences). All other processes were the same as the test groups.

### Opsonophagocytosis

Antibody-mediated bacterial uptake by macrophages was measured using the RAW264.7 cell line. RAW264.7 cells were seeded into 24-well plates and grown in Dulbecco’s Modified Eagle’s Medium (DMEM) containing 10% FBS (Newborn calf serum) and Pen/Strep at 37°C with 5% CO_2_ to confluence (5 x 10^5^ cells/well). Prior to infection, the old medium was replaced with fresh DMEM containing 10%FBS. The fresh wild-type *S*. Typhimurium and *S*. Paratyphi A bacteria were incubated with vaccinated mouse serum (10% in PBS) for 30 min on ice, and then added to the cell monolayer at a ratio of 10:1. After centrifugation (100 x g, 10 min), the 24-well plate containing the monolayer and opsonized bacteria was incubated at 37°C with 5% CO_2_ for 20–60 min. After incubation, extracellular bacteria were removed by replacing the old medium with fresh DMEM containing 10% FBS. Gentamicin was added to each well at a final concentration of 100 µg/ml, and the plates were incubated for another 1 h to kill non-internalized bacterial cells. The wells were sampled at time intervals of 0, 20, 40 and 60 min. After three washes with PBS, the macrophages were lysed with 1% Triton X-100, and internalized bacteria were enumerated on LB agar plates.

### Statistical analysis

Data were analyzed using GraphPad Prism 5 software package (Graph Software, San Diego, CA) by one-way or two-way ANOVA of variance followed by Tukey’s multiple-comparison posttest. The data were expressed as the means ± SEM. *P* < 0.05 was considered as significant difference.
