# Supplementary material for: Attenuated Salmonella Typhimurium expressing Salmonella Paratyphoid A O-antigen induces protective immune responses against two Salmonella strains
Source: Virulence. 2019 Jan 14;10(1):82–96. doi: 10.1080/21505594.2018.1559673 (PMC6363073; doi:10.1080/21505594.2018.1559673)
Supplement: Supplemental Material [file kvir-10-01-1559673-s001.zip › Supplementary Fig caption.docx]

## Supplementary Fig. S1. The O-antigen gene clusters of *S.* Paratyphi A and *S*. Typhimurium and their O-unit chemical structures.

The O-antigen gene clusters of *S*. Paratyphi A (A1, O2) and *S.* Typhimurium (B1, O4) were shown together with their chemical structures, revealing the sugar components and glycosidic linkages within their O-units. The immunodominant O-serotype factor was indicated in front of the gene cluster or the O-units. Genes were colored according to the synthesis pathways. Arrows represented the location and orientation of the genes. The types of glycosidic linkages were labelled in red. Sugar abbreviations: Abe, abequose; Par, paratose; L-Rha, L-rhamnose; D-Man, D-mannose; D-Gal, D-galactose; D-GlcNAc, D-acetylgalactosamine. Gene clusters were drawn to scale.

## Supplementary Fig. S2. Genetic manipulation and plasmid construction

(A) The allelic genes *abe*, *wzx*_B1_ and *wbaV*_B1_ in *S*. Typhimurium were subsequently deleted and replaced with *prt-tyv*_A1_, *wzx*_A1_ and *wbaV*_A1_ from S. Paratyphi A, respectively. (B) The *pagL* gene was deleted and replaced with an arabinose-regulated *abe* expression cassette. The *relA* gene was deleted and replaced with an arabinose-regulated *lacI* expression cassette. (C) Construction of plasmids pSS978. The recombinant *prt-tyv*_A1_, *wzx*_A1_ and *wbaV*_A1_ genes from S. Paratyphi A were under control of the P_trc_ promoter, which contained a *lac* repressor and was repressed by LacI. All inserted genes are drawn in black.

## Supplementary Fig. S3. Growth curves of O2 serotype converted mutants.

*In vitro* growth rates of *S.* Typhimurium mutants were measured by optical density measurements as an OD_600_ value at multiple time points.

## Supplementary Fig. S4. Attachment and invasion assays in Hep-2 cells.

The percentage of attachment and the invasion rate of *S*. Typhimurium mutants were evaluated as described in the Materials and Methods. The bacteria were added to each well at a multiplicity of infection (MOI) of 10:1. The error bars represent the standard errors of the means. “*”, *P*<0.05; “**”, *P*<0.01, vaccine strain versus S738.

## Supplementary Fig. S5. Colonization of murine Peyer’s patches, liver and spleen by live attenuated *S.* Typhimurium vaccines

All vaccine candidates were derived from the S738 parental strain. Colonization of Peyer’s patches (A), liver (B) and spleen (C) at 4 and 8 days post-inoculation is shown. The horizontal lines represent the means, and the error bars represented the standard errors of the means. “*”, *P*<0.05, vaccine strain versus S738.

## Supplementary Fig. S6. Serum IgG2a and IgG1 responses against *S*. Paratyphi A and *S*. Typhimurium LPS

The LPS of *S*. Paratyphi A and *S.* Typhimurium was used to coat ELISA plates. After a booster immunization, anti-*S.* Paratyphi A and anti-*S*. Typhimurium LPS serum IgG2a and IgG1 were measured in vaccinated mice by ELISA. (A) anti-*S*. Paratyphi A LPS IgG2a levels were significantly higher than those induced by S738 (**, *P*<0.01). Moreover, compared with the IgG2a responses, IgG1 levels were significantly lower in the serum from each vaccinated mice group (††, *P*<0.01). (B) anti-*S*. Typhimurium LPS IgG2a induced by S738 were significantly higher than that induced by S1112 (**, *P*<0.01). Moreover, the level of IgG1 was significantly lower than its corresponding IgG2a (††, *P*<0.01).The negative control groups (BSG) did not mount a detectable immune response in any test. The concentration of antibodies was calculated using a standard curve. All concentrations of the measured samples were within the range of the standard curve. Error bars represent the standard errors of the means.
