# Supplementary material for: Attenuated Salmonella Typhimurium expressing Salmonella Paratyphoid A O-antigen induces protective immune responses against two Salmonella strains
Source: Virulence. 2019 Jan 14;10(1):82–96. doi: 10.1080/21505594.2018.1559673 (PMC6363073; doi:10.1080/21505594.2018.1559673)
Supplement: Supplemental Material [file kvir-10-01-1559673-s001.zip › Supplementary Table S1 P2_11_19.docx]

**Table S1. Primers used in this work**

| **Primer name** | **Sequence 5'-3'** |
| --- | --- |
| Dabe-1F | gtggtctggctcagttgg |
| Dabe-1R | catgaaaatccagatagaataaattgaattagaaattcaaaccaaag |
| Dabe-2F | atttattctatctggattttcatgatcttttaataaat |
| Dabe-2R | gtttttggctctgcattctgatat |
| DwbaVB1-1F | gataggtgtcaatattttgcttggc |
| DwbaVB1-1R | aataacttactaatgattatcccttatttgccttaattaag |
| DwbaVB1-2F | ggataatcattagtaagttattatattgagattaaatgtag |
| DwbaVB1-2R | cctaaacgtgataggtttacgatc |
| D(abe-wzxB1)-1F | actgaattcctcgaattacc |
| D(abe-wzxB1)-1R | ataagcatatgacgcaatcaccagatagaataaattg |
| D(abe-wzxB1)-2F | ctggtgattgcgtcatatgcttatatcattttgtattccaac |
| D(abe-wzxB1)-1R | ctgcttgagaatcgagataagtttg |
| D(abe-wbaVB1)-1F | gaaacagggcttgcaatcttg |
| D(abe-wbaVB1)-1R | taacttactacgcaatcaccagatagaataaattg |
| D(abe-wbaVB1)-2F | ggtgattgcgtagtaagttattatattgagattaaatg |
| D(abe-wbaVB1)-2R | cgagtattgccacattccc |
| (G)Vec-Dabe-(prt-tyvA1)-F | actagttctatatgaatcgttaacaaattagtcgcgttatg |
| (G)Vec-Dabe-(prt-tyvA1)-R | ttcatcgcaatcaccagatagaataaattgaattagaaattc |
| (G)In_(prt-tyvA1)-F | tttattctatctggtgattgcgatgaaaattctaataatgggagcg |
| (G)In_(prt-tyvA1)-R | taatttgttaacgattcatatagaactagtccaatcatac |
| (G)Vec-Dabe-(prt-wzxA1)-F | aggctagcataatcatatgcttatatcattttgtattccaac |
| (G)Vec-Dabe-(prt-wzxA1)-R | tagaattttcatcgcaatcaccagatagaataaattg |
| (G)In_(prt-wzxA1)-F | tctggtgattgcgatgaaaattctaataatgggagcg |
| (G)In_(prt-wzxA1)-R | tataagcatatgattatgctagccttttactcttatac |
| (G)Vec_DwbaVB1_wbaVA1-F | atattttcatagtagtaagttattatattgagattaaatgtag |
| (G)Vec_DwbaVB1_wbaVA1-R | atgataccttcatatgattatcccttatttgccttaattaag |
| (G)In_wbaVA1-F | gggataatcatatgaaggtatcattttgtatcccaac |
| (G)In_wbaVA1-R | taacttactactatgaaaatattttttttattaccatttttgc |
| (G)Vec-Dabe-(prt-wbaVA1)-F | ttagaattttcatcgcaatcaccagatagaataaattg |
| (G)Vec-Dabe-(prt-wbaVA1)-R | aatattttcatagtagtaagttattatattgagattaaatgtag |
| (G)In_(prt-wbaVA1)-F | ctggtgattgcgatgaaaattctaataatgggagcg |
| (G)In_(prt-wbaVA1)-R | aataacttactactatgaaaatattttttttattaccatttttgc |
| (G)3700-F | aaacggttatgaggtaccgagctcgaattcgccctatagtg |
| (G)3700-R | atcgcaattcctctcgaggctagcccaaaaaaacg |
| (G)3700-abe-F  (G)3700-abe-R | ggctagcctcgagaggaattgcgatgacctttttgaaag  cgagctcggtacctcataaccgtttcagtagttcttc |
| (G)Vec-DpagL-(araCP_BAD_-abe)-F | gaaacggttatgaagttgaataacaattagcgagttg |
| (G)Vec-DpagL-(araCP_BAD_-abe)-R | atagaataataaatccaccaccatttcaatgtcaatag |
| (G)araCP_BAD_-abe-F | aatggtggtggatttattattctatcctagaattgtgataat |
| (G)araCP_BAD_-abe-R | tgttattcaacttcataaccgtttcagtagttcttc |
| (G)3337-F | atattttcatagggctgttttggcggatgagagaag |
| (G)3337-R | ttagaattttcatggtctgtttcctgtgtgaaattgttatcc |
| (G)3337-prt-wbaVA1-F | caggaaacagaccatgaaaattctaataatgggagcg |
| (G)3337-prt-wbaVA1-R | gccaaaacagccctatgaaaatattttttttattaccatttttgc |
