# Supplementary figures and images for: Attenuated Salmonella Typhimurium expressing Salmonella Paratyphoid A O-antigen induces protective immune responses against two Salmonella strains
Source: Virulence. 2019 Jan 14;10(1):82–96. doi: 10.1080/21505594.2018.1559673 (PMC6363073; doi:10.1080/21505594.2018.1559673)

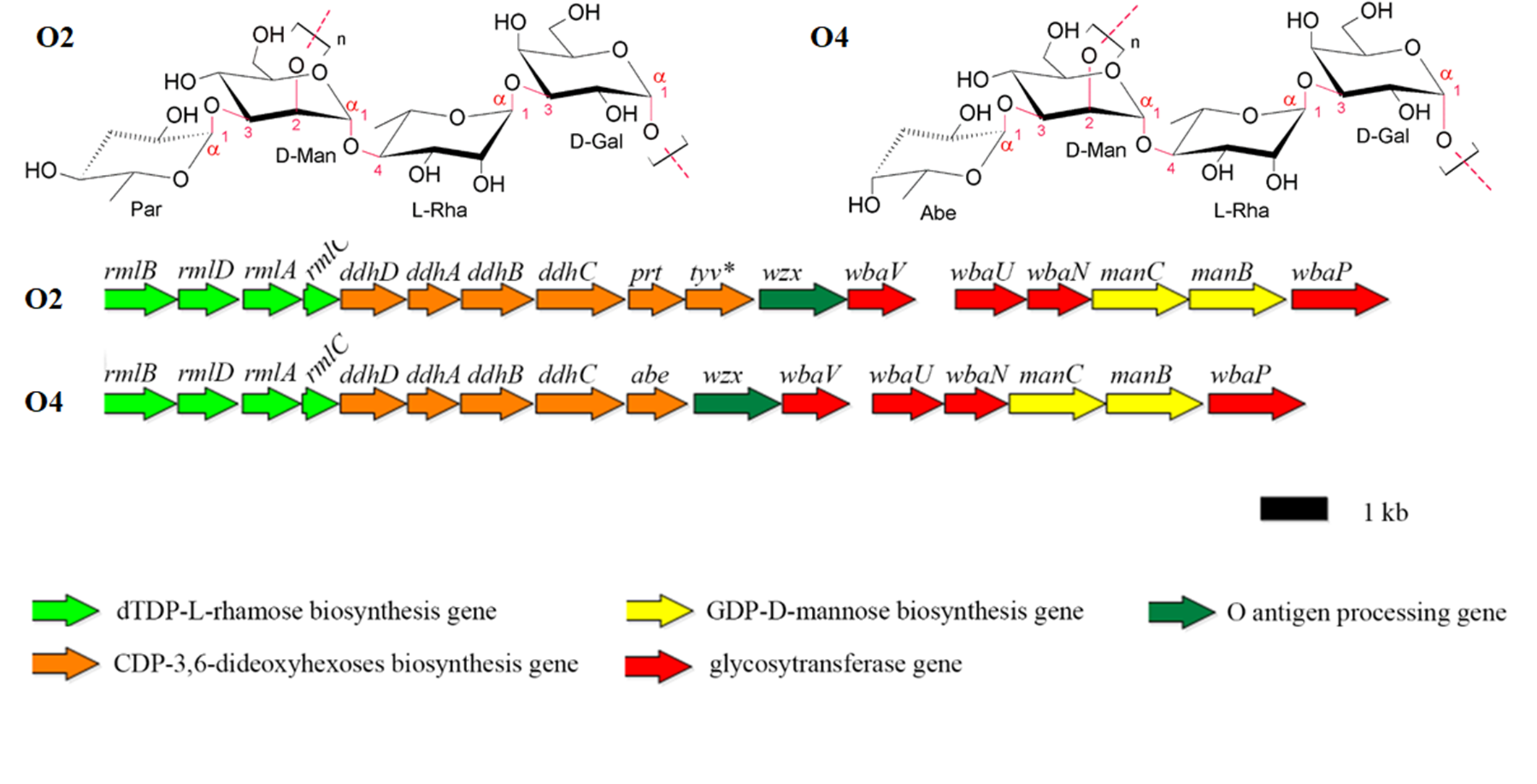

Supplement: Supplemental Material [file kvir-10-01-1559673-s001.zip › Figure S1_revised.tif]

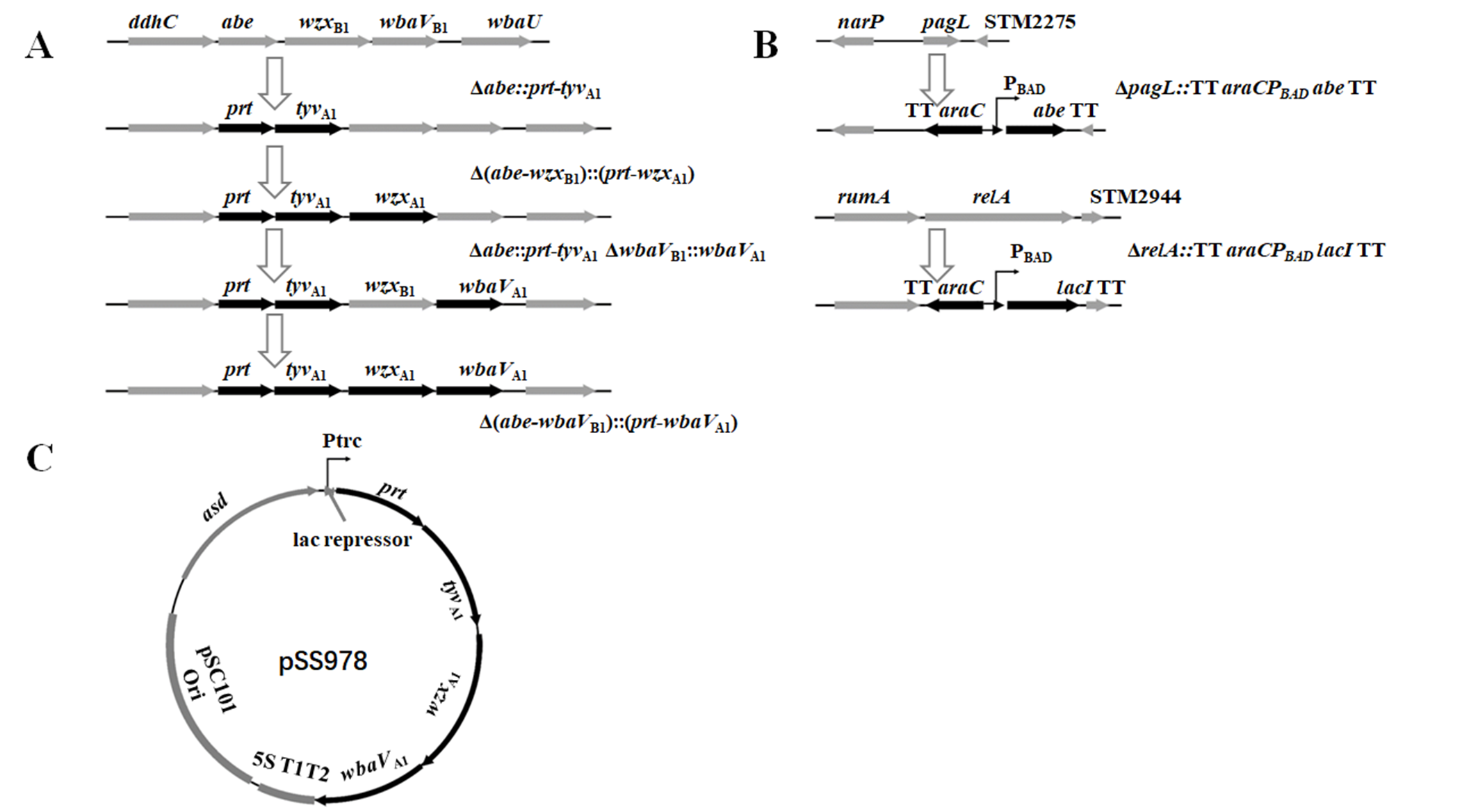

Supplement: Supplemental Material [file kvir-10-01-1559673-s001.zip › Figure S2_revised.tif]

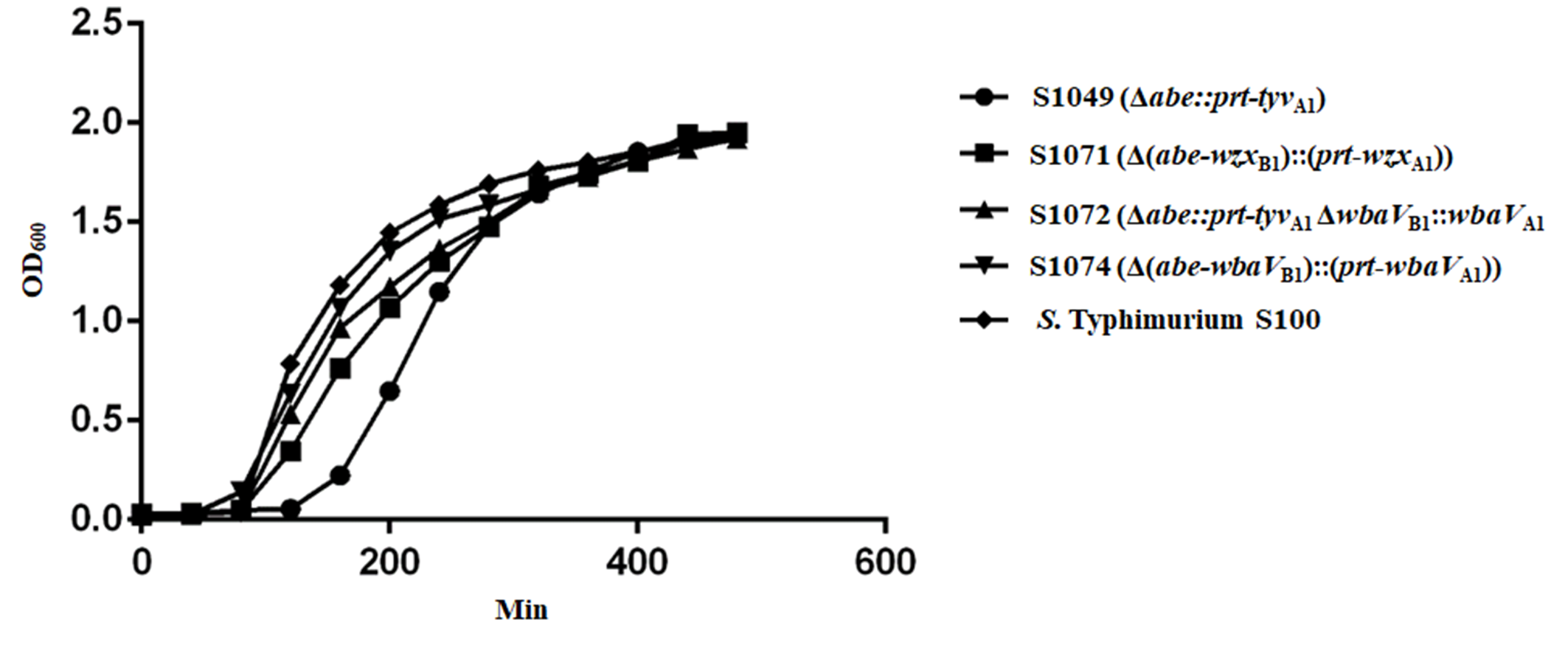

Supplement: Supplemental Material [file kvir-10-01-1559673-s001.zip › Figure S3_revised.tif]

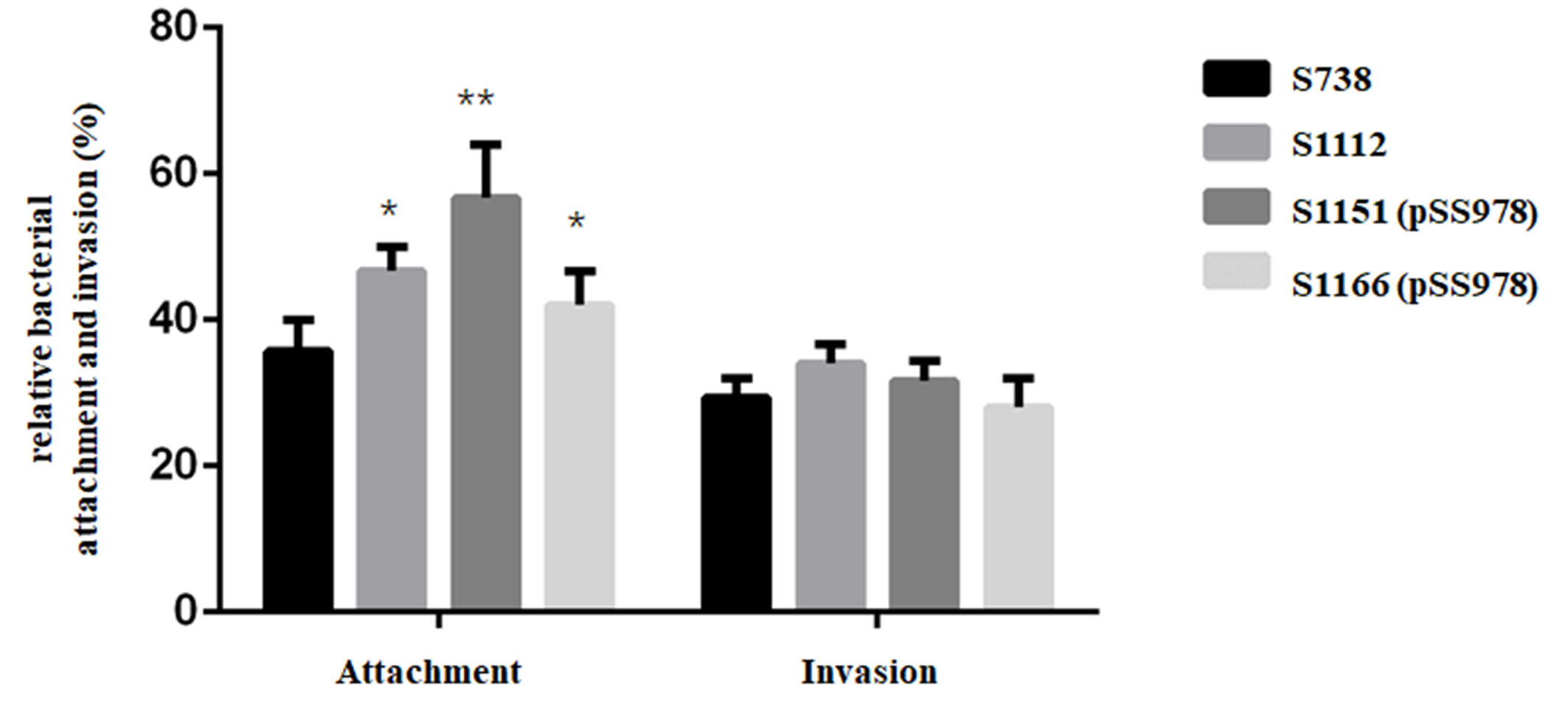

Supplement: Supplemental Material [file kvir-10-01-1559673-s001.zip › Figure S4_revised.tif]

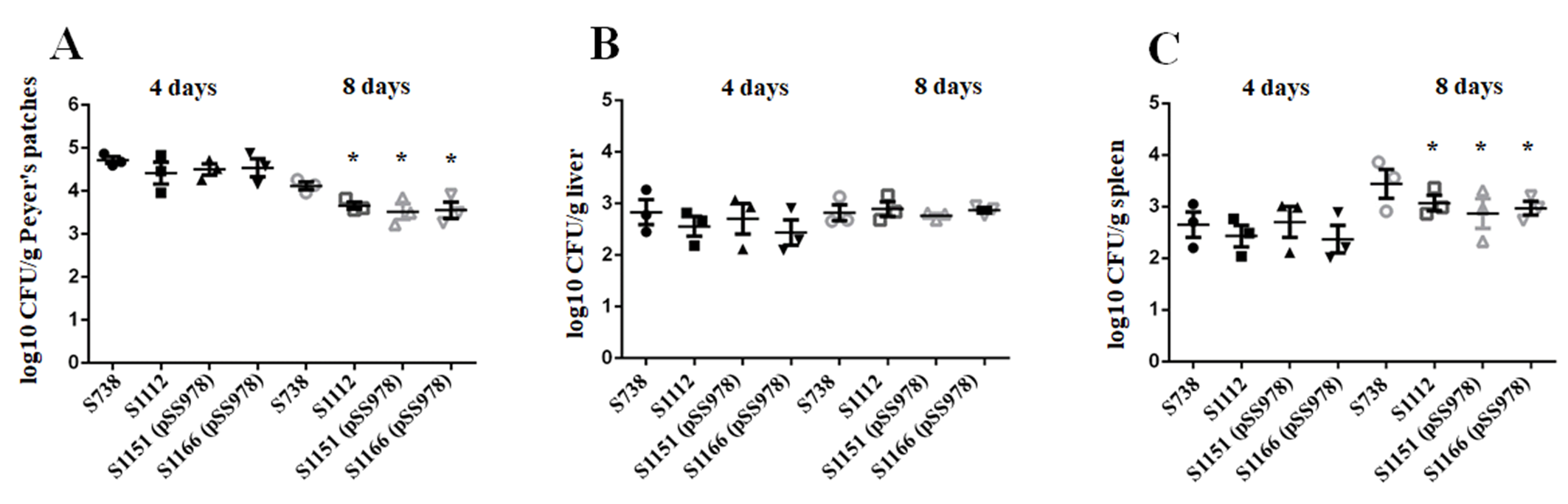

Supplement: Supplemental Material [file kvir-10-01-1559673-s001.zip › Figure S5_revised.tif]

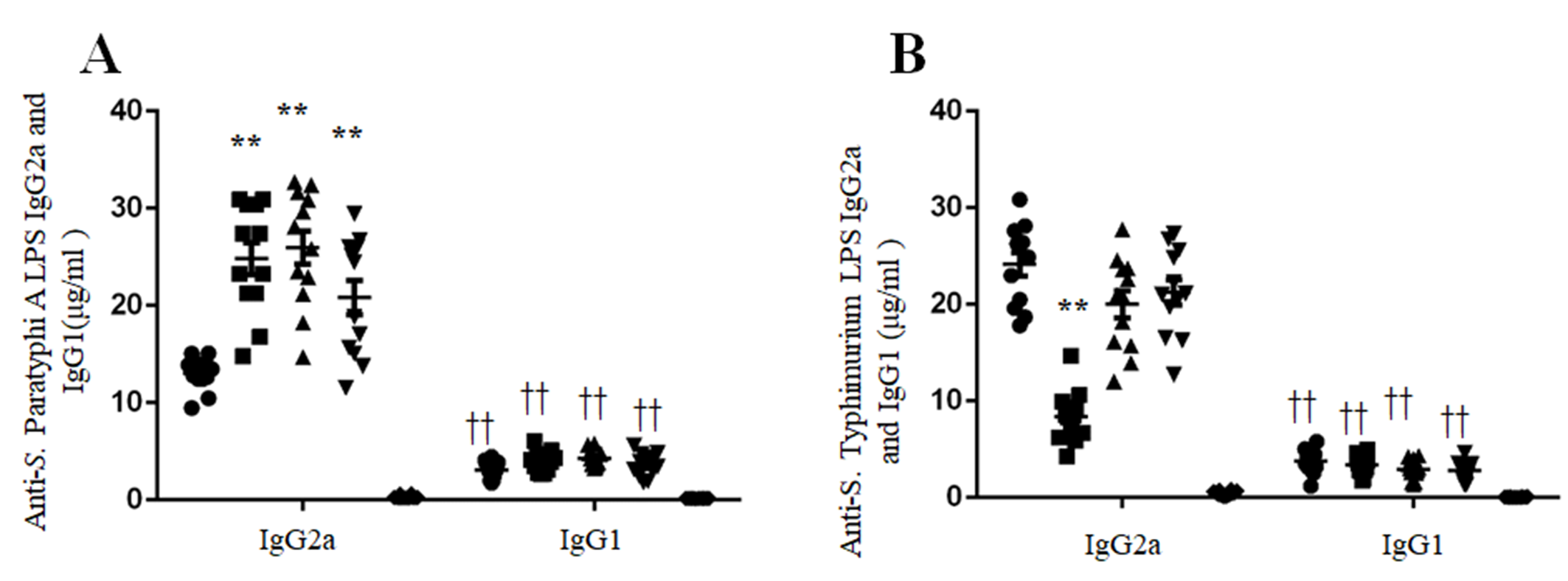

Supplement: Supplemental Material [file kvir-10-01-1559673-s001.zip › Figure S6_revised.tif]
